# Editorial: Hearing Loss and Cognitive Disorders

**DOI:** 10.3389/fnins.2022.902405

**Published:** 2022-05-16

**Authors:** Ke Liu, Wei Sun, Xiaoming Zhou, Shaowen Bao, Shusheng Gong, David Z. He

**Affiliations:** ^1^Department of Otolaryngology Head and Neck Surgery, Beijing Friendship Hospital, Capital Medical University, Beijing, China; ^2^Department of Communicative Disorders and Sciences, Center for Hearing and Deafness, The State University of New York at Buffalo, Buffalo, NY, United States; ^3^Key Laboratory of Brain Functional Genomics of Ministry of Education, Shanghai Key Laboratory of Brain Functional Genomics, School of Life Sciences, East China Normal University, Shanghai, China; ^4^Department of Physiology, University of Arizona, Tucson, AZ, United States; ^5^Department of Biomedical Sciences, Creighton University, Omaha, NE, United States

**Keywords:** hearing loss, cognitive disorders, behavior detection, cochlear implant, neuroimaging

Overall, 466 million people worldwide are estimated to be living with hearing loss. Most sensorineural hearing loss is caused by degeneration and loss of hair cells in the cochlea of the inner ear. Hair cells are specialized mechanoreceptors which transduce mechanical forces transmitted by sound to electrical activities (Hudspeth, 2014; Fettiplace, 2017). Hair cells in adult mammals are terminally differentiated and unable to regenerate once they are lost due to aging (Liu et al., 2022) or exposure to noise and ototoxic drugs (Garinis et al., 2021). Loss of hearing hinders the exchange of information, thus significantly impacting daily life and leading to social isolation and depression (Li et al., 2014). A case-control study first reported that hearing impairment in older adults was strongly associated with the likelihood of having dementia—raising the hypothesis that hearing impairment may contribute to dementia risk (Uhlmann et al., 1989). Recent epidemiologic and clinical research studies using both auditory and non-auditory cognitive tests have showed that hearing impairment is associated with a 30–40% rate of accelerated cognitive decline (Lin et al., 2013) and with a substantially increased risk of incident all-cause dementia (Lin et al., 2011; Gallacher et al., 2012). Compared to individuals with normal hearing, those people with a mild, moderate, and severe hearing impairment, respectively, had a 2-, 3-, and 5-fold increased risk of incident all-cause dementia over >10 years of follow-up (Lin et al., 2011). Neuroimaging studies have also demonstrated independent associations of hearing impairment with reduced cortical volumes in the auditory cortex (Peelle et al., 2011) and accelerated rates of lateral temporal lobe and whole brain atrophy (Lin et al., 2014).

Key challenges going forward pertain to both basic research on potential mechanisms as well as clinical issues to manage hearing loss. Several interesting questions need to be explored: (1) If hearing capacity of an individual has been deprived entirely since birth, how will this affect cognitive function? (2) If hearing is only partially deprived, will this still affect cognitive ability? (3) Will restoring hearing by wearing hearing aids or cochlear implants (CIs) also improve cognitive capacity? And (4) What is the neural basis underlying cognitive deficits caused by hearing impairment? In this Research Topic, 17 studies were accepted to address the questions above, at least partially. Thus, this Research Topic could provide new insights of the link between hearing loss and cognitive disorders and offer new evidence that supports the hypothesis that reduced sound input leads to impaired cognitive ability.

CIs are one of the most exciting auditory technological advancements which has brought great benefits to people with profound hearing loss. It remains unclear whether a CI can bring a significant change in cognition after hearing restoration. Several papers in this section focused on CI and cognition. Gao Q. et al. reviewed the literature on the developmental factors affecting speech perception of Mandarin-speaking children with CIs. The paper suggests that, compared with continuous improvements in speech perception in bilateral CI recipients, the outcome of speech perception in children with unilateral CIs is still not clear and requires more attention on their cognitive development. In this Research Topic, the study has investigated the mechanisms underlying visual-induced auditory interaction in pre-lingually deaf children (Wang J. et al.). Mirror mechanism can be considered as an activation of the mirror neuron which refers to a combined process of perception and execution. Their study examined the reliability of the visual-auditory interaction in pre-lingually deaf children with CIs and demonstrated that the CI-induced activation of mirror neurons could explain deficiencies in the classical cross-modal interaction.

Functional near-infrared spectroscopy imaging was used to assess the changes in neural activity in different brain regions after a CI. The study conducted by Wang Y. et al. shows that the cortical responses elicited by vocal emotional stimulation on the left pre-motor and supplementary motor area, the right middle temporal gyrus, and the right superior temporal gyrus are significantly different between preoperative and postoperative tests. Tian et al. reported that altered neural processing was associated with vocal emotional stimulation after CIs in a different study. Functional near-infrared spectroscopy imaging was used to investigate the cerebral representation of different sound sources. This study shows that the oxyhemoglobin response displays a different pattern of neural activities between the lateral and front sources in the auditory cortex and dorsolateral prefrontal cortex. Although CIs can restore and improve audibility in deaf children, it is not clear if children with CIs have impaired auditory sensory gating. Chen et al. carried out an investigation and showed that auditory sensory gating can develop during the early phase in children with CIs, however, long-term auditory deprivation may have a negative effect on sensory gating and attentional performance. Wang Q. et al. conducted a prospective cohort study on normal-hearing young adults who experienced measurable high-intensity noise exposure during a 1-day outdoor music festival. They show that the wave I amplitude of the auditory brainstem response decreases significantly after noise exposure. Their study provides evidence that cochlear synaptopathy may impair speech perception under a noise background.

Fu et al. conducted a study on the relationship between hearing loss and cognitive impairment in the elderly Chinese population. The study indicates that untreated age-related hearing loss (ARHL) can influence participants' performance during cognitive assessments. Xu et al. assessed the association of hearing acuity and cognitive function among a low-income elderly population in rural China. A total of 737 residents aged over 60 years were enrolled in this cross-sectional study. The study showed that hearing is associated with cognitive decline among older individuals. Another study by Diao et al. reported the correlation between hearing loss and cognitive decline in the elderly population. Gao J. et al. examined if middle ear surgery can affect cognitive ability and quality of life in patients with ARHL and chronic otitis media (COM). Interestingly, both the cognitive capacity and quality of life are improved after middle ear surgery. Guo P. et al. performed an functional MRI (fMRI) study on 45 children with congenital sensorineural hearing loss (CSNHL) to study the alteration of regional homogeneity. They found that children with CSNHL display regional homogeneity (ReHo) alterations in the auditory, visual, motor, and other related brain regions, suggesting a strong relationship between ReHo values and age involved in informatic integration and processing.

Animal models are a crucial tool to examine the mechanisms underlying cognitive disorders caused by hearing loss. This Research Topic also includes a number of papers that explored the connection of hearing loss with cognitive disorders using animal models. Liu et al. investigated how binaural cues of the primary auditory cortex are developed and affected by early reversible unilateral conductive hearing loss. They show that (1) the monaural response type, the binaural interaction type, and the distributions of the best interaural level differences (ILDs) in the auditory cortex display adult-like patterns shortly after hearing onset; (2) the developmental refinement of binaural processing can be disrupted by unilateral conductive hearing loss even in adulthood. These findings reveal the neuronal mechanism of the developmental refinements and plasticity in the perception of sound spatial location. To investigate the mechanism underlying ARHL and cognitive dysfunction, Deng et al. used the hippocampal transcriptome to study the correlations between impaired glutamatergic synapse pathway and ARHL. Their study shows that an impaired hippocampal glutamatergic synapse pathway is correlated with ARHL and cognitive dysfunction, and glutamine synthetase can serve as a crucial regulator in this process.

In this Research Topic, two studies provide some answers to the two questions we asked in the first paragraph. Li et al. examined time-dependent dynamic alterations in microglial activation and hippocampal neurogenesis after C57BL/6J mice were exposed to a single dose of broadband noise at 123 dB SPL. They show that the stress response and hearing loss may contribute to the alterations of microglia and hippocampal neurogenesis. In a different study, Guo R. et al. examined social memory in mice after complete elimination of peripheral auditory input before onset of hearing. They used a mouse model with complete hearing deprivation by deleting *Otof* . Their study reported a significant impaired social memory in the *Otof* knockout mice, suggesting that hearing is required for the formation of social memory. The illustration below shows the work of their study (Figure 1): wild-type mice exhibit normal behavioral features of social novelty, however, the mice with complete deprivation of hearing display impaired characteristics of social novelty; the diagram also provides a hypothesis of the behavioral impairment. Interestingly, their study shows that hearing loss may not affect the function of learning and memory. The authors excluded the possible influence of *Otof* deletion on the learning using immunostaining detection of otoferlin expression in the hippocampus.

**Figure 1:**
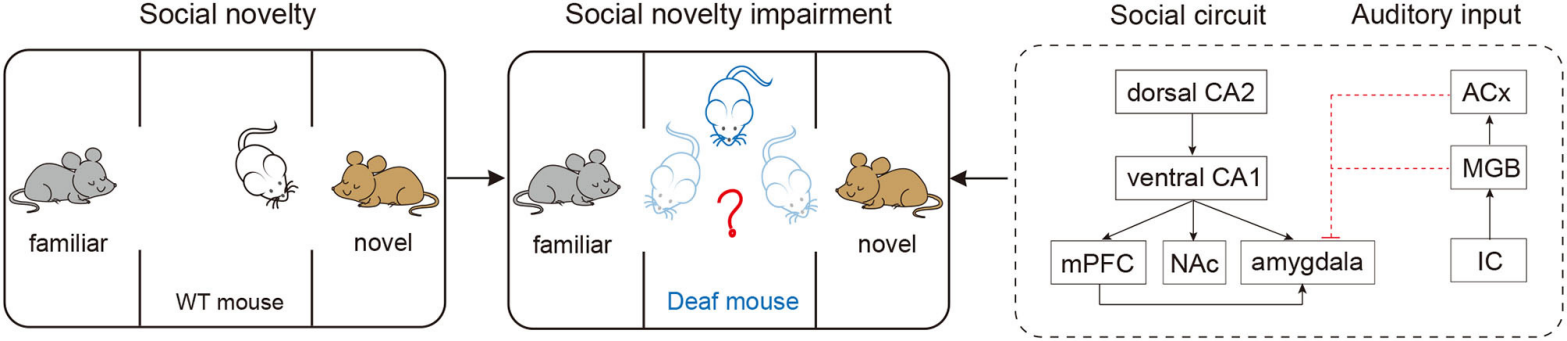
Complete elimination of peripheral auditory input before onset of hearing causes long-lasting impaired social memory in mice. Compared with the wild-type C57 mice (**left panel**, normal hearing), the otof^−/−^ mice spent significant reduced time in navigating the novelty **(middle panel)**, suggesting an impaired social memory. **Right panel**, A possible neuronal circuit responsible for the impaired social memory in deaf mice; both medial geniculate body (MGB) and auditory cortex (ACx) may have direct projection to amygdale (red dash lines), the projections can be blocked via complete hearing deprivation. CA1, field CA1 of hippocampus; CA2, field CA2 of hippocampus; mPFC, medial prefrontal cortex; NAc, accumbens nucleus; IC, inferior colliculus.

The serotonin transporter (SERT) greatly affects the activity of serotonergic neurons in the central nervous system. In this Research Topic, Pan et al. reported that SERT plays a key role in development and functional maintenance of auditory cortical neurons. Kwok Susanna et al. (2021) performed a review on pure tone audiometry and hearing loss in Alzheimer's disease. Based on the analysis of 248 published studies, they confirm the presence of poorer hearing ability in subjects with Alzheimer's disease. These data also suggest that ameliorating hearing loss could be an approach to mitigate the effects of Alzheimer's disease (Kwok et al.).

In summary, the 17 studies published in this Research Topic provide evidence on the connection between hearing loss and cognitive disorders. We are looking forward to seeing more evidence from prospective large-sample clinical trials on this topic, especially on the studies to test if hearing restoration *via* hearing aids and CIs could improve cognitive ability. In addition, we would also like to see more exciting basic science studies to elucidate the neuronal circuits that are responsible for cognitive disorders.

## Author Contributions

KL and WS contributed to the literature review and manuscript drafting. DH contributed to writing and revising the manuscript. XZ, SB, and SG wrote sections of the manuscript. All the authors contributed to the article and approved the submitted version.

## Funding

This work was supported by the National Natural Science Foundation of China (81830030, 82071037, and 32161160325), as well as the NIH grant R01 DC016807 from the NIDCD.

## Conflict of Interest

The authors declare that the research was conducted in the absence of any commercial or financial relationships that could be construed as a potential conflict of interest.

## Publisher's Note

All claims expressed in this article are solely those of the authors and do not necessarily represent those of their affiliated organizations, or those of the publisher, the editors and the reviewers. Any product that may be evaluated in this article, or claim that may be made by its manufacturer, is not guaranteed or endorsed by the publisher.
